# Case Report: Endometrial carcinoma harboring a rare *POLE* p.T278K variant and dual *MSH6* frameshift variants

**DOI:** 10.3389/fonc.2026.1964637

**Published:** 2026-10-15

**Authors:** Yuxuan Yang, Huimei Luo, Huixing Yan, Haoping Yu, Xinyue Ma, Qiuli Teng, Longxin Zhang, Jinwen Ye, Zeng Yuan, Qing Zhang

**Affiliations:** Department of Obstetrics and Gynecology, Qilu Hospital of Shandong University, Jinan, Shandong, China

**Keywords:** endometrial carcinoma, molecular classification, *MSH6*, *MSI-H*, p.T278K, *POLE*, *MLH1* promoter methylation

## Abstract

**Background:**

*POLE*-mutated endometrial carcinomas are associated with an excellent prognosis and have become an integral component of the molecular classification of endometrial carcinoma. However, the pathogenicity of many rare non-hotspot *POLE* exonuclease-domain variants remains uncertain, particularly when they occur in tumors harboring additional molecular alterations that may confound molecular classification.

**Methods:**

Histopathologic findings and immunohistochemical expression of mismatch repair proteins and p53 were evaluated. Targeted next-generation sequencing of tumor tissue was performed to characterize genomic alterations, microsatellite instability, tumor mutational burden, and the mutational spectrum. The non-hotspot *POLE* p.T278K variant was evaluated using the León-Castillo scoring framework. *MLH1* promoter methylation was assessed by methylation-specific PCR to further characterize the subclonal MLH1/PMS2 loss.

**Case summary:**

A 59-year-old woman with endometrioid endometrial carcinoma underwent total hysterectomy with surgical staging. Targeted next-generation sequencing identified a rare non-hotspot *POLE* exonuclease-domain variant, c.833C>A (p.T278K), together with two *MSH6* frameshift variants, c.3261del (p.F1088Sfs*2) and c.3261dup (p.F1088Lfs*5). The tumor demonstrated an ultramutated genomic profile with a tumor mutational burden of 370.0 mutations/Mb and an MSI-H phenotype. Immunohistochemistry demonstrated subclonal loss of MLH1, PMS2, and MSH6 with retained MSH2 expression; *MLH1* promoter methylation was also detected.

**Conclusion:**

This case adds clinicopathological and molecular evidence supporting the biological significance of the rare *POLE* p.T278K variant and illustrates a practical approach for interpreting uncommon *POLE* exonuclease-domain variants in endometrial carcinomas with multiple molecular classifiers. Accurate identification of the dominant molecular classifier is critical for appropriate molecular classification, prognostic assessment, and clinical management.

## 1 Introduction

The Cancer Genome Atlas (TCGA) established four molecular subgroups of endometrial carcinoma: POLE-ultramutated, microsatellite instability (MSI)-hypermutated, copy-number-low, and copy-number-high tumors (1). In routine clinical practice, these categories are approximated by the *POLE*-mutated (POLEmut), mismatch repair-deficient (MMRd), no specific molecular profile (NSMP), and p53-abnormal (p53abn) subgroups, respectively. This molecular classification has fundamentally changed the clinical management of endometrial carcinoma because it provides prognostic information beyond traditional clinicopathologic parameters and increasingly guides risk stratification and adjuvant treatment decisions (2, 3).

Pathogenic *POLE* exonuclease-domain variants are characterized by defective proofreading during DNA replication, resulting in an ultramutated genome with exceptionally high tumor mutational burden (TMB) and a characteristic mutational signature enriched for C>A and T>G substitutions (1, 4). Most pathogenic variants occur at recurrent hotspot residues, including p.P286R, p.V411L, p.S297F, p.A456P, and p.S459F. In contrast, interpretation of rare non-hotspot exonuclease-domain variants remains considerably more challenging because pathogenicity cannot be inferred solely from their location within the exonuclease domain. To address this issue, León-Castillo and colleagues developed an evidence-based scoring system that integrates TMB, mutational spectrum, insertion/deletion frequency, and recurrence data to distinguish pathogenic proofreading-deficient variants from variants of uncertain significance (5). More recently, analysis of a prospective cohort of 596 endometrial carcinomas demonstrated that several rare non-hotspot exonuclease-domain variants share the same ultramutated genomic characteristics as canonical hotspot mutations, supporting a biological continuum of *POLE*-driven hypermutation rather than a strictly hotspot-based classification (6).

An additional diagnostic challenge arises from the recognition that a small subset of endometrial carcinomas exhibits features of more than one molecular classifier, including concurrent POLE and MMR abnormalities (7). Current evidence indicates that tumors harboring pathogenic *POLE* mutations generally retain the favorable prognosis associated with the POLEmut subgroup, even in the presence of additional molecular alterations. However, whether this principle can be confidently extended to rare non-hotspot *POLE* variants remains uncertain because supporting clinicopathologic and molecular evidence is limited.

Here, we report an endometrioid endometrial carcinoma harboring the rare *POLE* exonuclease-domain variant p.T278K together with two *MSH6* frameshift variants, a microsatellite instability-high (MSI-H) phenotype, subclonal loss of MLH1, PMS2, and MSH6 with retained MSH2 expression, and *MLH1* promoter methylation. By integrating histopathologic findings, immunohistochemistry, targeted molecular profiling, and application of the León-Castillo scoring system, we provide additional evidence supporting a proofreading-deficient effect of *POLE* p.T278K and illustrate a practical approach to interpreting rare *POLE* variants in multiple-classifier endometrial carcinomas.

## 2 Case presentation

### 2.1 Chief complaints

A 59-year-old postmenopausal woman presented with a 6-day history of vaginal bleeding.

### 2.2 History of present illness

The patient developed postmenopausal vaginal bleeding on March 7, 2026, and presented to a local hospital the following day. Hysteroscopy with endometrial biopsy was performed, and histopathologic examination revealed endometrioid adenocarcinoma. She was subsequently referred to Qilu Hospital of Shandong University and admitted on March 13, 2026, for definitive surgical management. The clinical course is summarized in **Figure 1A**.

**Figure 1:**
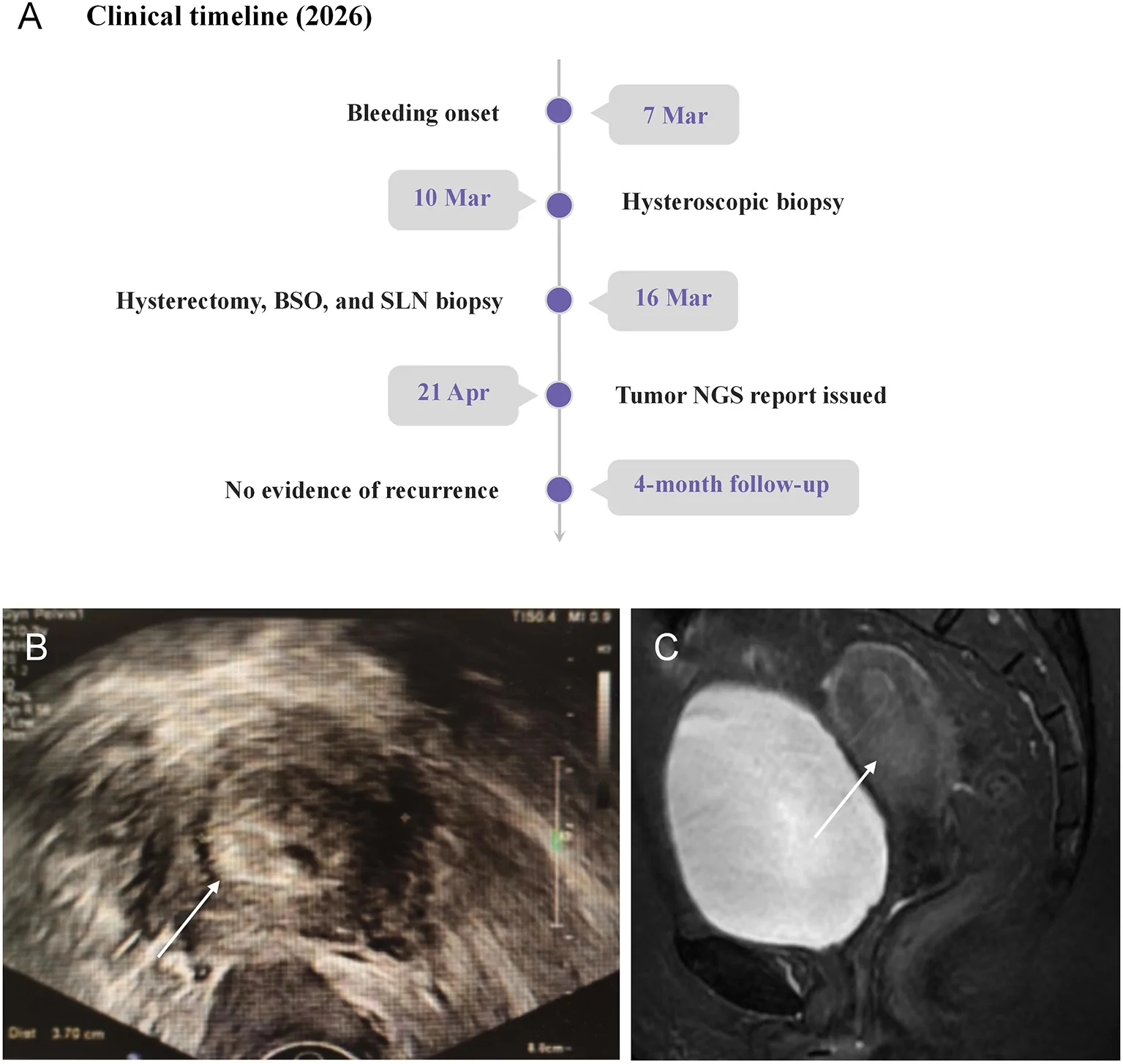
Clinical timeline and preoperative imaging. **(A)** Clinical timeline from bleeding onset to 4-month follow-up with no evidence of recurrence. **(B)** Transvaginal ultrasonography showing a hypoechoic mass within the uterine cavity (arrow). **(C)** Sagittal pelvic magnetic resonance imaging showing an endometrial mass filling the uterine cavity (arrow). BSO, bilateral salpingo-oophorectomy; NGS, next-generation sequencing; SLN, sentinel lymph node.

### 2.3 Past medical history

The patient had no significant past medical history.

### 2.4 Personal and family history

The patient had no family history of gynecologic or other hereditary malignancies, and there was no known history suggestive of a hereditary cancer syndrome.

### 2.5 Physical examination upon admission

Gynecologic examination demonstrated normally developed external genitalia. The vagina was patent and contained a small amount of bloody discharge. The cervix appeared grossly unremarkable, without cervical motion tenderness. The uterus was normal in size, anteverted, and mobile. No adnexal masses or other pelvic abnormalities were identified on bimanual examination.

### 2.6 Laboratory examinations

Serum CA125 was mildly elevated at 49.7 U/mL.

### 2.7 Imaging examinations

Transvaginal ultrasonography demonstrated an irregular endometrial mass measuring approximately 4.6 × 3.7 × 2.5 cm, with internal branching vascularity (**Figure 1B**). Pelvic magnetic resonance imaging revealed a 5.1 × 2.5 × 3.8 cm mass occupying the endometrial cavity. The lesion showed restricted diffusion, characterized by high signal intensity on diffusion-weighted imaging (DWI) and low apparent diffusion coefficient (ADC) values, consistent with a malignant neoplasm. No pelvic lymphadenopathy or pelvic free fluid was identified (**Figure 1C**).

### 2.8 Treatment

The patient underwent total hysterectomy, bilateral salpingo-oophorectomy, and bilateral pelvic sentinel lymph node biopsy.

Gross examination of the hysterectomy specimen revealed a 3.7 × 2.5 cm exophytic endometrial tumor. On cut section, the tumor was gray-white, firm, and measured 3.7 × 1.4 cm. It invaded approximately 20% of the myometrial thickness without involvement of the lower uterine segment or cervix.

## 3 Materials and methods

### 3.1 Immunohistochemistry

Immunohistochemistry was performed on formalin-fixed, paraffin-embedded tumor sections according to the manufacturers’ protocols. Primary antibodies included ER (SP1), PR (1E2), HER2 (4B5), p16 (E6H4), and Ki-67 (30-9) (Ventana/Roche, USA); p53 (DO-7), MLH1 (ES05), PMS2 (EP51), MSH2 (RED2), and MSH6 (EP49) (ZSGB-BIO, China); and D2-40 (D2-40; Maixin, China). Histopathological and immunohistochemical findings were independently reviewed by two pathologists, with discrepancies resolved by consensus.

### 3.2 Molecular analyses

DNA from FFPE tumor tissue underwent amplicon-based molecular typing and 572-gene hybrid-capture sequencing at AmoyDx (Xiamen, China). Tumor-cell content was estimated on a corresponding H&E-stained section. Libraries were sequenced on an Illumina NextSeq 550 platform. Reads were aligned to hg19 and analyzed using the Human Cancer Polygene Mutation Analysis software (AmoyDx).

The 572-gene panel served as the primary assay for variant reporting, MSI, TMB, and nucleotide-substitution analysis. Sequencing quality metrics included a Q30 rate of 94.76%, target coverage of 99.53%, and mean effective depths of 1509.79× in hotspot regions and 704.15× in non-hotspot regions. MSI was assessed at 55 loci by the molecular-typing assay and 116 loci by the primary assay, with an MSI-score cutoff of 200 for the latter. Laboratory-reported TMB was expressed as base substitutions and indels per megabase.

Laboratory-filtered coding SNVs, including synonymous substitutions, and small coding indels were included; noncoding variants were excluded. Substitution proportions were calculated among the included coding SNVs, whereas the indel proportion was calculated among all included coding SNVs and small indels. Variant nomenclature followed HGVS recommendations, and clinical significance was assessed according to the 2017 AMP/ASCO/CAP framework. The POLE p.T278K locus was manually reviewed in the primary-assay BAM file using Integrative Genomics Viewer (IGV).

As an ancillary analysis, *MLH1* promoter methylation was assessed in FFPE tumor tissue by methylation-specific polymerase chain reaction (MS-PCR) at Jinan KingMed Diagnostics Laboratory (Jinan, China), with the result qualitatively classified as methylated or unmethylated according to the laboratory-validated protocol.

The mutational spectrum was compared with the *POLE*-ultramutated profile described by TCGA (1). The León-Castillo *POLE* scoring framework, developed from whole-exome sequencing data, was used for exploratory assessment (5).

## 4 Results

### 4.1 Pathology and immunohistochemical results

Histologic examination demonstrated a FIGO grade 2 endometrioid endometrial carcinoma. The tumor invaded less than one-half of the myometrium. There was focal lymphovascular space invasion (LVSI), but no involvement of the uterine serosa, cervix, or parametrial tissues. Bilateral pelvic sentinel lymph nodes were negative for metastatic carcinoma (left, 0/2; right, 0/1). The fallopian tubes were unremarkable, and both ovaries showed age-related atrophy. The final pathologic stage was pT1aN0(sn) (FIGO 2009 stage IA).

Immunohistochemically, the tumor cells showed diffuse expression of estrogen receptor (ER, 2+, approximately 80%) and progesterone receptor (PR, 2+, approximately 80%). HER2 immunostaining was scored as 2+, while p53 demonstrated a wild-type staining pattern. Diffuse p16 expression and a Ki-67 proliferation index of approximately 60% were present. D2–40 immunostaining highlighted focal lymphovascular invasion.

Mismatch repair (MMR) immunohistochemistry demonstrated subclonal loss of MLH1, PMS2, and MSH6, with retained MSH2 expression (**Figure 2**). Ancillary MS-PCR detected *MLH1* promoter methylation.

**Figure 2:**
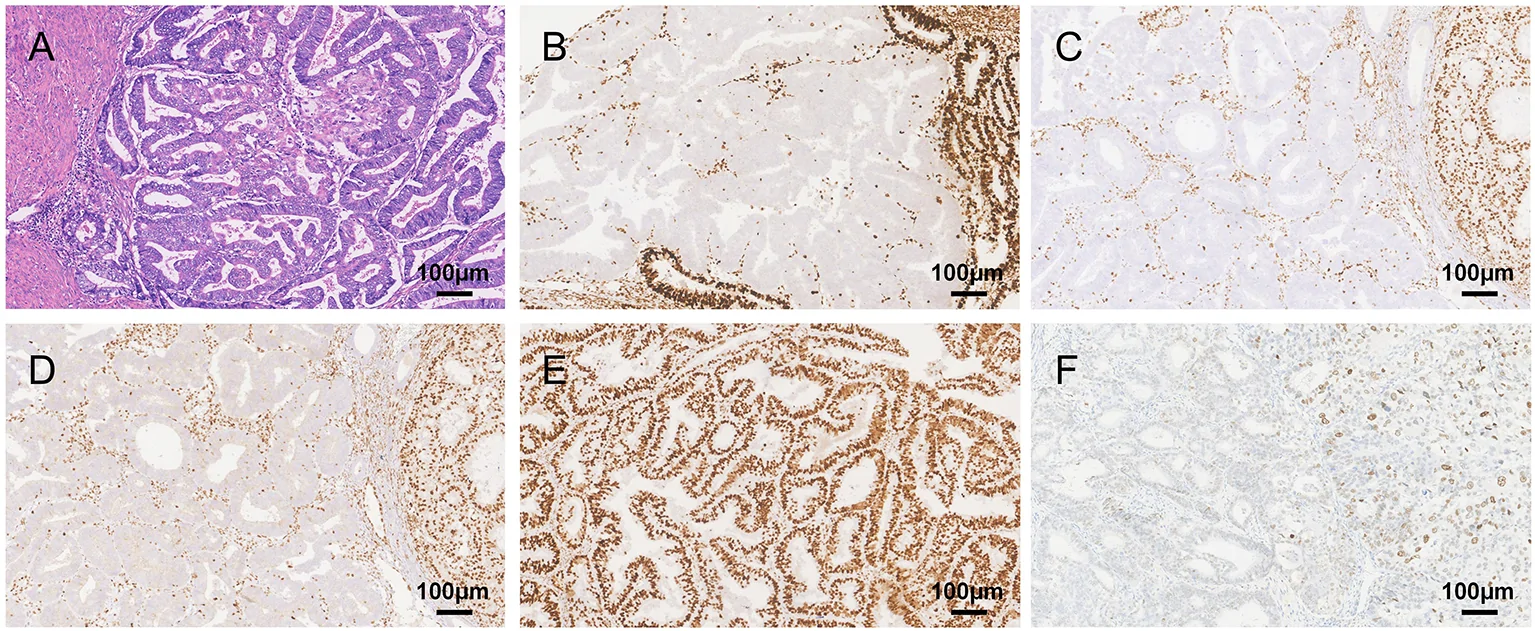
Histopathological and immunohistochemical findings in the endometrial carcinoma. **(A)** H&E staining; **(B)** MSH6; **(C)** MLH1; **(D)** PMS2; **(E)** MSH2; **(F)** p53.

### 4.2 MSI and TMB results

NGS-based microsatellite analysis demonstrated a microsatellite instability-high (MSI-H) phenotype, with an MSI score of 1279.88, substantially exceeding the predefined positivity threshold of 200. The tumor also exhibited an ultramutated genomic profile, with a tumor mutational burden (TMB) of 370.0 mutations/Mb.

### 4.3 Molecular findings

Targeted next-generation sequencing (NGS) was performed on formalin-fixed, paraffin-embedded tumor tissue containing approximately 45% tumor cells, as independently confirmed by two pathologists. Hybrid-capture sequencing identified a rare *POLE* exonuclease-domain variant, NM_006231.4:c.833C>A (p.Thr278Lys), located in exon 9, with a variant allele frequency (VAF) of 40.42% (**Figure 3A**). In addition, the *MSH6* variants NM_000179.3:c.3261del (p.Phe1088Serfs*2) (VAF 17.64%) and c.3261dup (p.Phe1088Leufs*5) (VAF 34.94%) were also detected (**Figure 3B**). IGV review of the primary-assay BAM file showed 440 reads at chr12:133,253,208 (hg19), of which 187 supported the alternate T allele (42.5%; 88 forward and 99 reverse reads) (**Figure 3A**).

**Figure 3:**
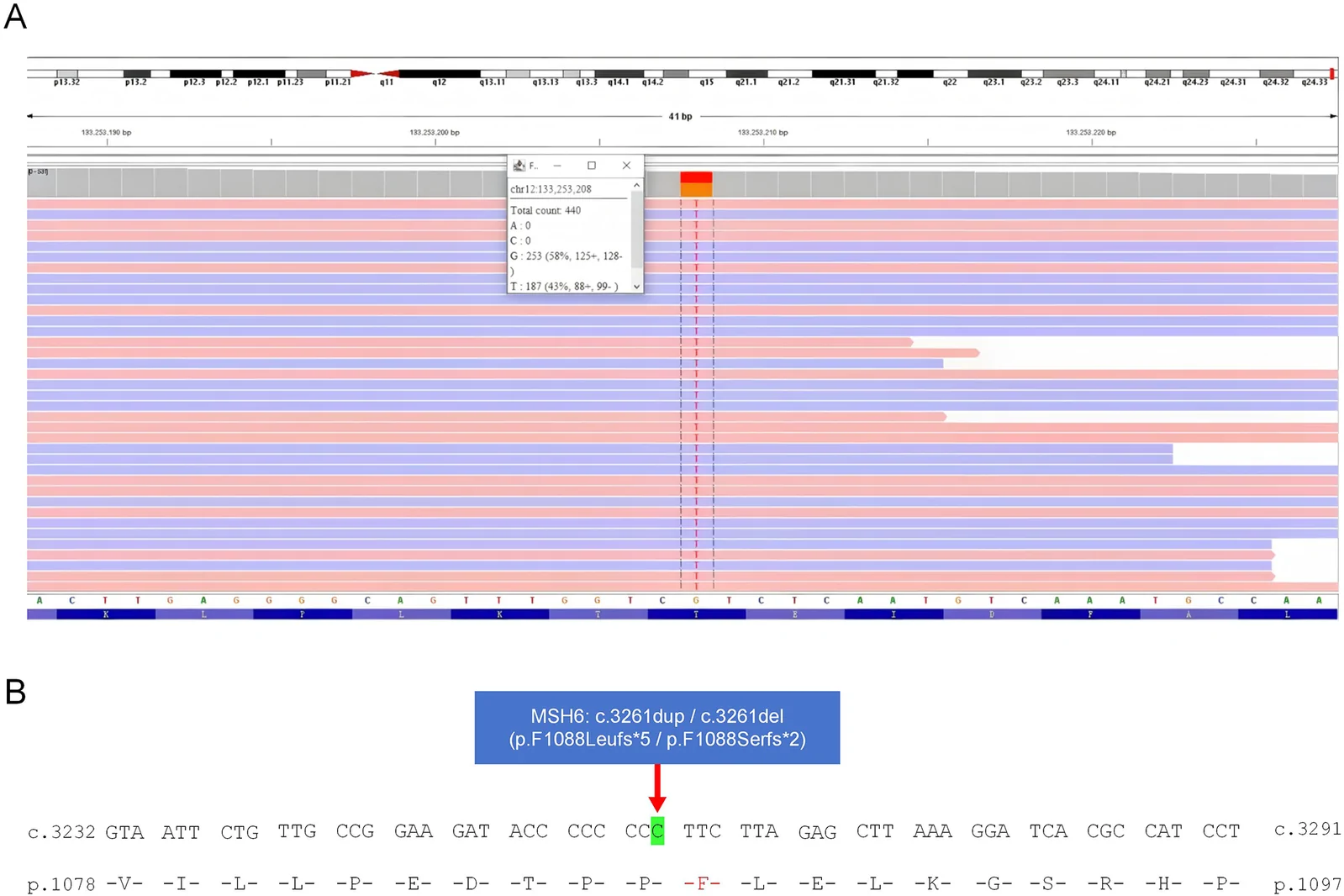
**(A)** IGV visualization of the *POLE* c.833C>A (p.Thr278Lys) variant, supported by 187 of 440 reads (42.5%). **(B)** Schematic representation of the *MSH6* c.3261dup and c.3261del frameshift variants.

Additional alterations were identified in several genes frequently mutated in endometrial carcinoma, including *PTEN*, *PIK3CA*, *ARID1A*, *PPP2R1A*, and *FGFR2*, together with variants involving multiple DNA damage response genes, including *BRCA2*, *ATM*, *ATR*, and *RAD54L*. A *TP53* missense variant, NM_000546.6:c.844C>T [p.(Arg282Trp)], exon 8, was also detected (VAF 32.37%), despite a wild-type p53 immunohistochemical staining pattern.

Because sequencing was performed using tumor tissue without matched normal DNA, the somatic or germline origin of the *POLE* and *MSH6* variants could not be definitively determined.

Exploratory application of the León-Castillo *POLE* scoring system yielded a score of 4, with one point each assigned for TMB (370.0 mutations/Mb), C>A (26.35%), T>G (8.87%), and C>G (0.25%); no point was assigned for the indel proportion (9.17%) or recurrence, as p.T278K was absent from the TCGA endometrial carcinoma cohort and was represented by only one endometrial carcinoma record in COSMIC (**Table 1**). The ultrahigh TMB and C>A-enriched substitution profile were consistent with the general features of the POLE-ultramutated subgroup described by TCGA (1).

**Table 1:** Exploratory application of the León-Castillo *POLE* scoring framework (5).

Criterion	Results	*POLE* score
C > A ≥20%	26.35%	1
T > G ≥4%	8.87%	1
Indels ≤5%	9.17%	0
C > G ≤0.6%	0.25%	1
TMB >100 muts/Mb	370.00 mutations/Mb	1
Recurrent variant in EC	No	0
Total *POLE* score		4

### 4.4 Outcome and follow-up

The patient received no adjuvant therapy following surgery. At 4 months of follow-up, serum CA125 levels remained within the normal range, pelvic ultrasonography showed no evidence of disease, and there was no clinical or ultrasonographic evidence of tumor recurrence.

## 5 Discussion

This case illustrates the diagnostic challenge of interpreting a rare non-hotspot *POLE* exonuclease-domain variant in an endometrial carcinoma exhibiting features of multiple molecular classifiers. The tumor demonstrated an ultramutated phenotype, MSI-H, and subclonal loss of MLH1, PMS2, and MSH6 with retained MSH2 expression. *MLH1* promoter methylation was also detected. Collectively, these findings support classification of the tumor as a POLEmut/MMRd multiple-classifier endometrial carcinoma (5, 7).

The principal molecular finding was the rare non-hotspot *POLE* exonuclease-domain variant p.T278K. Before the present report, p.T278K had been documented in two patients with endometrial carcinoma: one detailed case reported by Cui et al., which exhibited an ultramutated phenotype and a *POLE* score of 4, and one additional patient identified in a prospective cohort of 596 sequenced endometrial carcinomas (6, 8). The variant lies within the Exo I motif, close to the catalytic residue E277 and within the DNA-binding cleft. A yeast-based reversion assay demonstrated a mutator phenotype associated with p.T278K, providing functional evidence of impaired proofreading activity (9). In the present tumor, the ultrahigh TMB and characteristic substitution profile yielded an exploratory León-Castillo *POLE* score of 4. Because this framework was developed using whole-exome data, its application to targeted-panel findings should be regarded as exploratory and supportive rather than definitive. Taken together, the molecular phenotype of the present tumor, prior clinical observations, and published functional evidence provide converging, although not definitive, support for a proofreading-deficient effect of *POLE* p.T278K.

The MMR-deficient phenotype may involve more than one molecular mechanism. *MLH1* promoter methylation provides a plausible molecular correlate for the subclonal MLH1/PMS2 loss, whereas the two *MSH6* frameshift variants may account for subclonal MSH6 loss. Together with the MSI-H phenotype, these findings support a true MMR-deficient component. Previous microdissection-based studies demonstrated that regional *MLH1* promoter methylation can correspond to subclonal MLH1/PMS2 loss; however, the bulk-tumor assay used here cannot establish spatial correspondence (10). A recent study reported a POLE-mutated endometrial carcinoma with *MLH1* promoter methylation and identified another POLE-ultramutated TCGA case with MLH1 silencing (11). To our knowledge, the present case therefore appears to represent the third reported example of an endometrial carcinoma with co-occurring *POLE* alteration and *MLH1* promoter methylation.

The two *MSH6* frameshift variants, p.F1088Sfs*2 and p.F1088Lfs*5, result from reciprocal indels at the same c.3261 position within a low-complexity sequence and are predicted to cause protein truncation. The sequence context, unequal variant allele frequencies, and proofreading-deficient phenotype are compatible with secondary or parallel somatic events arising through replication slippage (12, 13). However, both variants are classified in ClinVar as pathogenic in the germline context (Variation IDs 89363 and 89364), and tumor-only sequencing cannot exclude a germline origin. Peripheral-blood germline testing was therefore recommended. Regardless of their origin, these variants do not alter the tumor’s classification as a POLEmut/MMRd multiple-classifier endometrial carcinoma, with POLEmut taking precedence in molecular classification and prognostic interpretation (5, 7). The discordance between *TP53* sequencing and p53 immunohistochemistry may reflect intratumoral heterogeneity, differences in p53 protein regulation, or technical factors, although the specific mechanism remains unresolved. In the context of the POLEmut/MMRd phenotype, this discordance did not alter the final molecular classification or postoperative management.

Classification of the tumor as POLEmut refined its molecular classification and prognostic interpretation. Under the 2023 FIGO molecular staging system, the tumor was classified as stage IAmPOLEmut (14). However, even without definitive assignment to the POLEmut subgroup, its grade 2 endometrioid histology, <50% myometrial invasion, focal LVSI, negative lymph nodes, and MMRd status would meet the FIGO 2023 IA2 criteria and fall within the low-risk category. Therefore, omission of adjuvant therapy was also consistent with the 2025 ESGO–ESTRO–ESP guidelines (15). MMRd/MSI-H status remains clinically relevant should recurrent or advanced disease develop because it may inform the use of immune checkpoint inhibitors (15).

The principal limitations were tumor-only sequencing, targeted-panel rather than whole-exome analysis, qualitative *MLH1* promoter methylation testing of bulk tumor tissue, absence of formal mutational signature analysis, and short follow-up.

In summary, this case expands the limited clinical evidence on *POLE* p.T278K and highlights the importance of integrating mutational phenotype, MMR findings, and *MLH1* promoter methylation status when evaluating rare *POLE* variants and multiple-classifier endometrial carcinomas.

## Patient perspective

I was very satisfied with the overall diagnostic and treatment process. My doctors clearly explained the purpose of molecular testing and discussed the postoperative management and follow-up plan with me. I felt well informed and was able to participate in decisions about my care.

## Data Availability

The original contributions presented in the study are included in the article/supplementary material, further inquiries can be directed to the corresponding author/s.

## References

[1] Levine DA The Cancer Genome Atlas Research Network . Integrated genomic characterization of endometrial carcinoma. Nature. (2013) 497:67–73. doi: 10.1038/nature12113 23636398 PMC3704730

[2] Church DN Stelloo E Nout RA Valtcheva N Depreeuw J Ter Haar N . Prognostic significance of POLE proofreading mutations in endometrial cancer. J Natl Cancer Inst. (2015) 107:dju402. doi: 10.1093/jnci/dju402 25505230 PMC4301706

[3] León-Castillo A De Boer SM Powell ME Mileshkin LR Mackay HJ Leary A . Molecular classification of the PORTEC-3 trial for high-risk endometrial cancer: Impact on prognosis and benefit from adjuvant therapy. JCO. (2020) 38:3388–97. doi: 10.1200/JCO.20.00549 32749941 PMC7527156

[4] Church DN Briggs SEW Palles C Domingo E Kearsey SJ Grimes JM . DNA polymerase ε and δ exonuclease domain mutations in endometrial cancer. Hum Mol Genet. (2013) 22:2820–8. doi: 10.1093/hmg/ddt131 23528559 PMC3690967

[5] León‐Castillo A Britton H McConechy MK McAlpine JN Nout R Kommoss S . Interpretation of somatic *POLE* mutations in endometrial carcinoma. J Pathol. (2020) 250:323–35. doi: 10.1002/path.5372 31829442 PMC7065171

[6] Nero C Trozzi R Persiani F Rossi S Mastrantoni L Duranti S . *POLE* mutations in endometrial carcinoma: Clinical and genomic landscape from a large prospective single‐center cohort. Cancer. (2025) 131:e35731. doi: 10.1002/cncr.35731 39865420 PMC11771542

[7] León‐Castillo A Gilvazquez E Nout R Smit VT McAlpine JN McConechy M . Clinicopathological and molecular characterisation of ‘multiple‐classifier’ endometrial carcinomas. J Pathol. (2020) 250:312–22. doi: 10.1002/path.5373 31829447 PMC7065184

[8] Cui J Chen X Zhai Q Chen N Li X Zhang Y . A novel somatic mutation in POLE exonuclease domain associated with ultra-mutational signature and MMR deficiency in endometrial cancer: A case report. Diagn Pathol. (2023) 18:19. doi: 10.1186/s13000-023-01287-y 36765365 PMC9912575

[9] Castellsagué E Li R Aligue R González S Sanz J Martin E . Novel *POLE* pathogenic germline variant in a family with multiple primary tumors results in distinct mutational signatures. Hum Mutat. (2019) 40:36–41. doi: 10.1002/humu.23676 30362666

[10] Pai RK Plesec TP Abdul-Karim FW Yang B Marquard J Shadrach B . Abrupt loss of MLH1 and PMS2 expression in endometrial carcinoma. Am J Surg Pathol. (2015) 39:993–9. doi: 10.1097/PAS.0000000000000415 25786082

[11] Flaus A Cui Z Miladinovic M Yoon J-Y . Pitfalls in MLH1 promoter methylation assessment, including POLEmut/MLH1meth endometrial adenocarcinoma. Pathol Res Pract. (2026) 277:156303. doi: 10.1016/j.prp.2025.156303 41297483

[12] Hodel KP Sun MJS Ungerleider N Park VS Williams LG Bauer DL . POLE mutation spectra are shaped by the mutant allele identity, its abundance, and mismatch repair status. Mol Cell. (2020) 78:1166–1177.e6. doi: 10.1016/j.molcel.2020.05.012 32497495 PMC8177757

[13] Haradhvala NJ Kim J Maruvka YE Polak P Rosebrock D Livitz D . Distinct mutational signatures characterize concurrent loss of polymerase proofreading and mismatch repair. Nat Commun. (2018) 9:1746. doi: 10.1038/s41467-018-04002-4 29717118 PMC5931517

[14] Berek JS Matias‐Guiu X Creutzberg C Fotopoulou C Gaffney D Kehoe S . FIGO staging of endometrial cancer: 2023. Int J Gynaecol Obstet. (2023) 162:383–94. doi: 10.1002/ijgo.14923 37337978

[15] Concin N Matias-Guiu X Cibula D Colombo N Creutzberg CL Ledermann J . ESGO–ESTRO–ESP guidelines for the management of patients with endometrial carcinoma: Update 2025. Lancet Oncol. (2025) 26:e423–35. doi: 10.1016/S1470-2045(25)00167-6 40744042

